# Oral biofilms exposure to chlorhexidine results in altered microbial composition and metabolic profile

**DOI:** 10.1038/s41522-020-0124-3

**Published:** 2020-03-20

**Authors:** Ioanna Chatzigiannidou, Wim Teughels, Tom Van de Wiele, Nico Boon

**Affiliations:** 1 Center for Microbial Ecology and Technology, Coupure Links 653, 9000 Gent, Belgium; 2 Department of Oral Health Sciences, KU Leuven, Kapucijnenvoer 33, 3000 Leuven, Belgium

**Keywords:** Dentistry, Antimicrobials, Biofilms

## Abstract

Oral diseases (e.g., dental caries, periodontitis) are developed when the healthy oral microbiome is imbalanced allowing the increase of pathobiont strains. Common practice to prevent or treat such diseases is the use of antiseptics, like chlorhexidine. However, the impact of these antiseptics on the composition and metabolic activity of the oral microbiome is poorly addressed. Using two types of oral biofilms—a 14-species community (more controllable) and human tongue microbiota (more representative)—the impact of short-term chlorhexidine exposure was explored in-depth. In both models, oral biofilms treated with chlorhexidine exhibited a pattern of inactivation (>3 log units) and fast regrowth to the initial bacterial concentrations. Moreover, the chlorhexidine treatment induced profound shifts in microbiota composition and metabolic activity. In some cases, disease associated traits were increased (such as higher abundance of pathobiont strains or shift in high lactate production). Our results highlight the need for alternative treatments that selectively target the disease-associated bacteria in the biofilm without targeting the commensal microorganisms.

## Introduction

The oral microbiome is one of the most diverse microbial communities that inhabit the human body^1^. More than 700 bacterial taxa have been identified to date which inhabit different niches in the oral cavity by forming biofilms on surfaces such as the teeth, the gingiva or the tongue (www.homd.org). The oral microbiome is in continuous interaction with environmental factors and its host. Under homeostatic conditions, the oral microbiome is stable and in symbiosis with its host^2,3^. However environmental perturbations can lead to a shift into dysbiotic biofilms which can be a causative factor of oral diseases, such as caries and periodontitis^4,5^.

Control of the oral microbiome, prevention and treatment of oral diseases is often achieved with the help of antimicrobials, such as antibiotics and antiseptics. The use of antimicrobials aims at decreasing the total microbial load to tackle the disease. One of the most common antiseptics used in oral health care is chlorhexidine (CHX), a bactericidal agent. CHX has a broad spectrum efficacy and acts by interfering with the cytoplasmic or inner bacterial wall once it has successfully crossed the outside membrane^6^. CHX is commonly added in treatment products at a concentration of 0.12 or 0.2%. Both concentrations are well above the minimum inhibitory concentration (MIC) of tested oral strains^7^. Yet, such MIC tests expose the microorganisms to a constant concentration of the biocide. Although this approach is a good proxy for systemic treatment, it is not representative of a topical treatment such as a mouth rinse application. In reality the contact time between the bacteria and the antiseptic is between 60 and 90 s during oral treatment. Moreover in the case of oral diseases, the target is a polymicrobial biofilm and bacteria in biofilms exhibit increased tolerance to antimicrobials compared to planktonic bacteria^7^. Finally, the biofilm architecture can greatly influence the outcome of the treatment. The outside layers of the biofilm are more susceptible to the antiseptic compared to the inner layers^8^.

All the above highlight the need for adequate and representative models to study oral biofilms and their response to current or future treatments. These models need to capture the complexity of the biofilm communities but at the same time need to offer controllability and reproducibility. In the field of oral microbiology, synthetic communities consisting of two to six key oral strains have been extensively used to study the interactions between oral microorganisms and their response to external stimuli^9–14^. These simpler synthetic communities allow for a well-controlled system with known players. Their inter-species interactions can be more easily studied and modeled^15^. Furthermore, species concentrations can be determined accurately. On the opposite side of the experimental spectrum saliva or plaque samples have been used to grow poly-microbial biofilms in vitro^16,17^. These more complex communities better capture the diversity of an in vivo oral biofilm and the interactions between the oral microorganisms. It is a step closer to a more realistic model. Yet, the increased complexity of these systems leads to reduced controllability and require more elaborate methods to track the community response and dynamics. As a result, studies assessing the effect of antiseptics on in vitro biofilms from saliva or plaque origin have primarily focused on microscopic techniques^8,18^. Such an approach studies the response of a biofilm as one unit and does not examine the community composition. Only few studies have tried to resolve the above by using amplicon sequencing to track community shifts^19^. However, the outcome of the treatment might be greatly influenced by the composition of the surviving community, making it very important to identify it while studying the effect of the antimicrobial stress on the polymicrobial biofilm.

The aim of the present study was to evaluate the impact of antiseptic treatment on in vitro oral biofilms. Previous studies have shown that mouthwash rinsing, even when applied consecutively for many days, had only a temporal effect^20^. We hypothesized that the treatment would not only affect the living cells concentration but also the composition and metabolic activity of the surviving community, and that consecutive exposures could enhance this phenomenon. We used two different microbial communities to mimic the polymicrobial oral biofilm: (i) a 14-strain biofilm and (ii) a tongue swab derived microbiota biofilm. The biofilms were exposed for a short (5 min) period to 0.12% CHX every 24 h to simulate the oral care procedure and we dynamically monitored the cell viability, community composition, and metabolic activity.

## Results

A dynamic in vitro model was used to mimic oral biofilms and study their response to antiseptic stress. A 14-species synthetic community or a tongue swab from four subjects was used as an inoculum and the response to CHX was evaluated in terms of microbial survival and regrowth, community composition, and metabolic activity.

### CHX leads to initial drop in biofilm bacterial cell concentration followed by quick recovery

To test the effect of the antiseptic treatment on cell viability, in vitro oral biofilms were treated for 5 min with 0.12% CHX during three consecutive days. Microbial survival was determined by means of flow cytometry and SGPI (viability) staining. The cells were separated in three clusters based on their flow cytometric profile: intact, damaged, and dead.

**Fig. 1:**
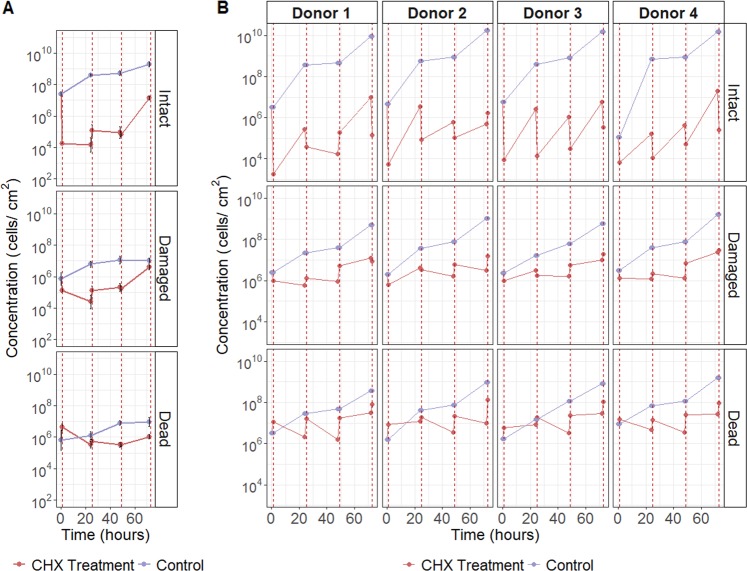
Biofilms exhibited a pattern of kill and regrowth after treatment with 0.12% CHX. Concentrations of live, damaged, and dead cells for **a**. 14-species biofilms. Points are the average of four replicates and error bars represent the standard deviation. **b** For tongue deriving microbiota biofilm from four individuals (donors). The red line is the concentration of CHX treated biofilms, purple/blue for the non-treated control biofilms. The vertical red lines represent the points of treatment.

The initial concentration of intact cells in the 14-species biofilms was ~10^7^ cells/cm^2^. Control biofilms that were not exposed to CHX exhibited a constant growth over time to a final concentration of 10^11^ intact cells/cm^2^ at the end of the experiment. In contrast, the first short exposure to CHX resulted in a large decrease of intact cells with three log units (to 10^4^ cells/cm^2^), yet without completely inactivating the biofilm (Fig. 1a). As expected, an increase in damaged and dead cells was observed after the treatment. The intact cell concentration remained stable for the following 24 and 48 h despite the two additional CHX treatments. At 72 h, after three consecutive days of short CHX exposures, however, the bacterial concentration sharply rose to 10^7^ cells/cm^2^ (similar to the concentration at 0 h) (Fig. 1a). The more complex tongue-swab derived biofilms displayed a similar pattern of inactivation and regrowth (Fig. 1b). However their regrowth response was even faster and took already place after 24 h. More in detail, the initial intact cell concentration was 10^6^ cells/cm^2^ and dropped to 10^4^ cells/cm^2^ immediately after exposure to CHX. Yet, the following 24 h of incubation displayed a 2 log regrowth. The following CHX exposures at 48 and 72 h showed similar patterns: a drop in intact cell concentration immediately after CHX treatment, followed by a rapid regrowth in the subsequent incubation period, eventually bringing back the intact cell concentration to the original value of 10^7^ cells/cm^2^. This pattern was consistent for all incubations with human derived oral biofilms. As expected, the dynamics of damaged and dead cell concentrations were the opposite of those from the intact cells.

### Biofilm composition shift after CHX treatment

We hypothesized that the antiseptic treatment alters the microbial composition of the biofilm, as it is already known that susceptibility to CHX is strain dependent. To determine how the composition changed over time, the abundances of the different taxa were measured either by means of qPCR (for the 14 strain-biofilm) or by 16S rRNA gene amplicon sequencing (for the tongue microbiota derived biofilms).

**Fig. 2:**
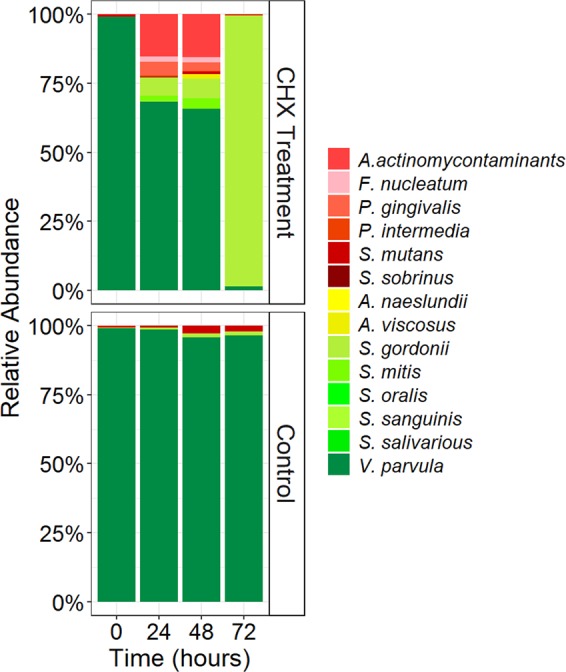
The bacterial composition of the 14-strains biofilms over the course of treatment. Relative abundance of the individual strains in 14-strain biofilms every 24 h and before the next CHX treatment. The percentages are the average of four replicates.

Non-treated 14 strains biofilms were highly dominated by *Veillonella parvula* (99% relative abundance, Fig. 2). In contrast, CHX exposure had a big impact on community composition as *V. parvula* relative abundance rapidly decreased to >5%, while *Streptococcus gordonii* showed a big increase in abundance reaching 94% (±0.08) of the total biofilm at 72 h. The six pathobionts (*P. gingivalis, P. intermedia, A. actinomycontaminants, F. nucleatum, S. mutans*, and *S. sobrinus*) were present at 3–4 log concentrations lower in the biofilms compared to the more dominant strains (*A. actinomycontaminants* <10^4^ cells/cm^2^ while *P. gingivalis, P. intermedia, F. nucleatum, S. mutans*, and *S. sobrinus* were <10^3^ cells/cm^2^). However, these concentrations were unaffected by CHX and thus remained stable over the course of the treatment.

**Fig. 3:**
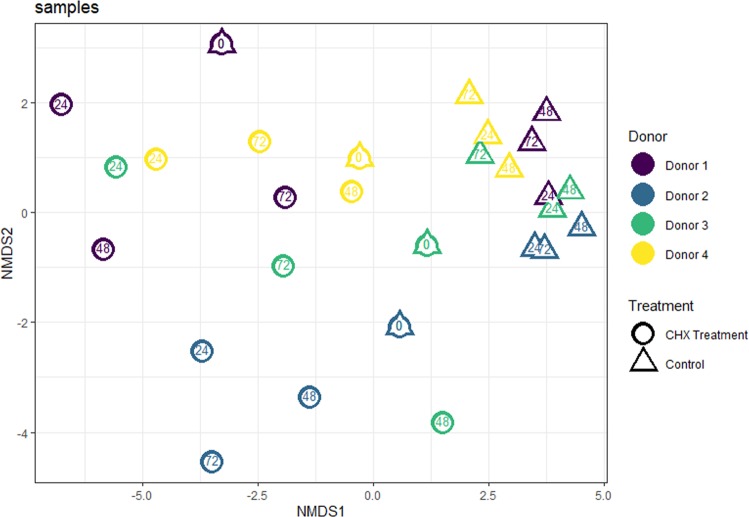
nMDS plot representing the β-diversity of the tongue deriving microbiota biofilm samples based on Bray Curtis dissimilarity index. Samples deriving from different donors have a different color, circles are for CHX treated samples, while triangles symbolize non-treated control samples. The number indicates the time of the sampling in hours.

16S rRNA gene amplicon sequence analysis revealed the initial composition of the tongue-swab derived biofilms to be donor-dependent (Fig. 3). This inter-individual difference became less clear under control conditions as β-diversity of mature biofilms dropped over time with samples clustering together in non-metric multidimensional scaling (NMDS) ordination plots based on Bray–Curtis dissimilarities (Fig. 3). In sharp contrast, tongue-swab derived biofilms exposed to CHX were diverging from each other over time: a clear donor-dependent effect was observed.

**Fig. 4:**
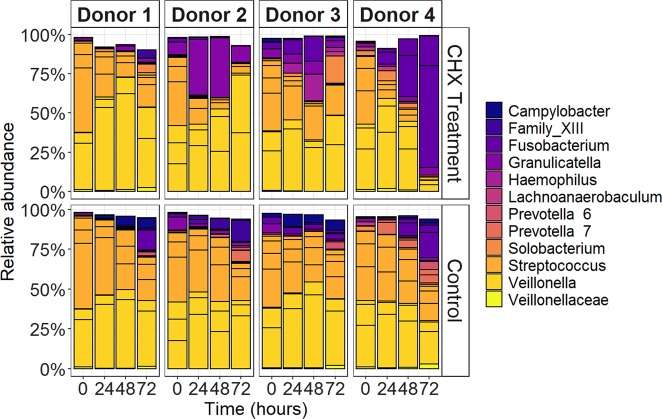
The bacterial composition of oral biofilms over the course of treatment. Relative abundance of the 20 most abundant OTUs for the tongue derived microbiota biofilms every 24 h and before the next CHX treatment.

At baseline all tongue microbiota derived biofilms were dominated by *Veillonella* and *Streptococcus*, 84–97% were classified as Veillonellaceae and Streptococcaceae (Fig. 4). Over the course of the treatment donor-dependent changes were observed in the most abundant taxa. Donor 1-derived biofilms exhibited a small decrease in relative abundances of *Veillonella*. Donor 2-biofilm displayed a large shift in the composition after the first CHX treatment with an increase in the relative abundance of the genus *Granulicatella* after the first two treatments. Relative abundances of *Fusobacterium*, *Haemophilus* and *Solobacterium* increased during the course of treatment in donor 3-biofilm. Finally, donor 4-biofilm exhibited the most profound community shift. The composition shifted towards a *Fusobacterium* monodominance with this genus accounting for more than 90% of the total community.

### Metabolic activity shifted after CHX treatment reflecting the community shift

To investigate the effect of antiseptic stress on the metabolic activity of the community, the ability to produce or consume organic acids was evaluated. The concentration of the lactate, acetate, propionate, formate and butyrate on surrounding medium was measured every 24 h.

**Fig. 5:**
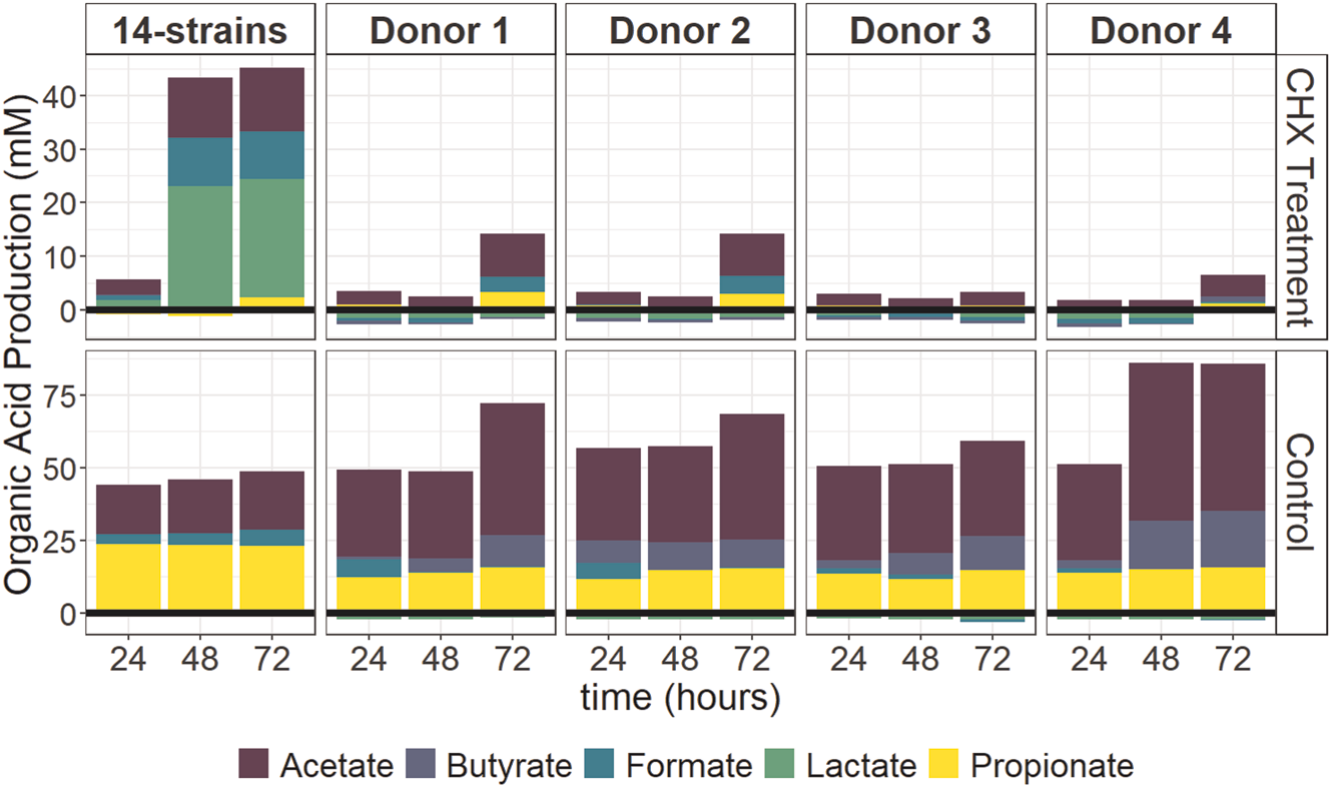
The effect of CHX treatment on the metabolic activity of the in vitro oral biofilms. The organic acid production or consumption by the 14-strains (average of four replicates) and tongue-deriving microbiota biofilms in between the daily treatments with 0.12% CHX.

Non-treated 14-strains biofilms produced propionate (18.96 ± 0.6 mM/day), acetate (13.49 ± 0.19 mM/day) and formate (2.83 ± 0.68 mM/day) after the first 24 h (Fig. 5). The relative production of organic acids remained constant over time in maturing (non-treated) biofilms. In contrast, total organic acid production by CHX-treated biofilms greatly decreased to 4.76 ± 0.36 mM/day after the first exposure to antiseptics, compared to non-treated biofilms, which produced 43.15 ± 0.33 mM/day. After the initial decrease, total organic acid production recovered but with a different composition, shifting to a high production of lactate (23.1 ± 1.00 mM).

Non-treated in vitro tongue microbiota biofilms produced acetate (25.35 ± 1.34 mM/day), propionate (10.24 ± 1.00 mM/day) and lower concentrations of formate (3.01 ± 2.4 mM/day) and butyrate (2.96 ± 2.87 mM/day) (total 49.84 ± 3.2 mM/day). Total production increased over time (69.06 ± 11.38 mM/day) as well as the relative concentration of butyrate (accounting for 14–23%). Small differences were observed between donors. On the other hand, CHX-treated samples produced much lower concentrations of organic acids (8.03 ± 5.62 mM/day). Notably the donor effect was again obvious. For donor 1 and 2 relative production did not change with acetate and propionate being the organic acids produced at higher concentrations. However, in donor 4-biofilm a higher production of butyrate (+1.5 mM/day compared to the other donors) was observed. These results correspond with the community composition shift and the high relative abundances of *Fusobacterium* in the same biofilm.

## Discussion

Oral biofilms are polymicrobial communities with a vital role in oral and systemic health. Oral care commonly includes the use of wide spectrum antimicrobials (i.e., chlorhexidine). However, these non-targeted treatments can have a big impact on the innate commensal microbial community. In fact, previous studies suggest that antiseptic stress affects oral biofilm composition and metabolic activity^19^. Meanwhile the long-term effect and dynamic response from the oral biofilms to antiseptics is poorly understood. In this study we investigated the response of in vitro oral biofilms to consecutive treatments with a wide spectrum antiseptic, i.e., CHX. We evaluated the impact from short daily CHX exposures on microbiota composition and activity with two model systems for oral biofilms: (i) a biofilm derived from a synthetic community composed of 14 commensal and pathobiont oral strains, being more controllable because of its defined composition and (ii) a set of biofilms derived from tongue swab microbiota from healthy individuals, being a closer proxy to the complexity of the in vivo microbiota. Although moderate model-dependent differences in the biofilm response to CHX exposure were noted, the overall trend of CHX affecting community composition and functionality was consistent in both model systems.

Simulating a mouthwash procedure, short daily exposures to 0.12% CHX exhibited a repeated pattern of inactivation and rapid regrowth in both biofilm types. Although chlorhexidine exhibits a high substantivity which means that the antimicrobial action will continue longer than the actual rinse^21^, our findings show CHX to only have a temporal effect on oral bacterial biofilms which is supported by both in vivo and in vitro previous studies^18,22^. These results indicate that oral antiseptics will fall short of keeping microbial numbers under control and are thus ineffective in maintaining oral hygiene. Moreover, as broad-spectrum antiseptics such as CHX also affect the endogenous oral microbiota, there is an increased risk that microbial dysbiosis in the oral cavity will occur resulting in the development of oral diseases. Indeed, our results confirm that the community composition from both synthetic as natural oral biofilms are drastically altered upon repeated CHX exposure. The 14 species-biofilms displayed profound shifts from a *V. parvula* dominated community (99%) to a *Streptococcus* dominated community, more specifically *Streptococcus gordonii* (98%) upon CHX treatment. The higher tolerance of *S. gordonii* to CHX, as exhibited by a CHX susceptibility test (MIC), may explain this result (Supplementary Table 2). *Veillonella* species are common colonizers of the oral cavity and are able to produce propionate from lactate^23^, which is typically produced by *Streptococcus* species. It was therefore not a surprise that the observed phylogenetic shifts coincided with drastic changes in metabolite profiles. Untreated biofilms mainly produced propionate (23.3 mM) while CHX exposed biofilms mainly produced lactate (23.1 mM ± 1) (Fig. 5). *Streptococcus gordonii*, the most abundant strain in the CHX treated 14-strain biofilms, is considered a primary colonizer of the dental surface^24^ and produces l-lactate as primary metabolite. While it is less aciduric than mutans streptococci^25^, it succeeded in dominating the synthetic oral biofilm despite the significant decrease in pH from 7 to pH 5.8 during the incubation experiment. High concentrations of lactate in combination with a low pH (the pKa of lactic acid/lactate being 3.86) are important determinants of tooth demineralization and tooth decay and increase the risk for dental caries. At the same time, the concentrations of the pathobiont strains were not reduced (with the exception of *S. mutans*). This outcome cannot be explained by the individual tolerance to chlorhexidine as most strains exhibit similar or lower MIC than other strains which relative abundance decreased drastically (e.g., *V. parvula*). The higher survival rates of the pathobiont strains could be explained by the biogeography of the oral biofilm, where these strains normally inhabit the inner layers of the biofilm and they are thus more protected^26^.

The bacterial community of the tongue microbiota biofilms was also affected by CHX treatments. The most pronounced shift was observed in the biofilms derived from donor 4 where there was a continuous increase of the relative abundance of the genus *Fusobacterium* over the course of the daily CHX treatments. The increased dominance of *Fusobacterium* is significant because it is considered a bridge organism between early and late colonizers in oral biofilms facilitating biofilm maturation and attachment of pathobionts. This genus is prevalent in periodontal plaque samples^27,28^ and thus linked to periodontal disease. The observed higher production of butyrate by the CHX exposed natural biofilm is also indicative of higher *Fusobacterium* dominance^23,29^. Butyrate is a bacterial metabolic by-product with detrimental effects for oral health, triggering inflammation in gingival fibroblasts^30^ and disrupting the gingival epithelial barrier^31^. With respect to the other in vivo derived biofilms, we found the antiseptic treatment to impact the biofilms in a dynamic and donor-dependent manner, with composition and metabolic activity shifting after every treatment. Despite the fact that non-treated mature biofilms clustered together according to β-diversity, the community composition of CHX treated biofilms was clearly dependent on initial inoculum. Our results confirm previous observations where inter-personal microbiome variability was described as one of the main drivers in response to oral treatment^32,33^.

Overall our findings made evident that wide spectrum antimicrobials cannot guarantee a shift to a healthy state. On the contrary they can further perturb the commensal microbiome. These findings are in accordance with recent in vivo study that shows that CHX treatment although kept microbial load in lower numbers than no treatment, did so by unselective targeting of the oral microbiome which resulted in higher relative abundances of several periodontitis related taxa (i.e., *Fusobacterium*)^34^. Moreover antiseptics have been already shown to increase pathogenic characteristics^35^, resistance to antiseptics^36^, and cytotoxicity to host cells^37^. For this reason there is a clear need for alternative approaches that do not indiscriminately target the oral microbiome but would specifically and selectively target pathogenic strains^38^ (or their virulence—virulence therapy) and promote or re-establish a healthy microbiome (pro-biotics and pre-biotics)^39–41^.

To conclude, we state that antiseptics are inadequate as sole treatment of oral biofilms. We observed that short treatment with 0.12% CHX, a concentration commonly used in oral care products, only temporarily decreased the viable cell concentration. Furthermore, our results suggest that initial microbiome composition highly influenced the outcome of the antiseptic treatment with disease associated characteristics increasing after treatment in certain cases. Further in vivo experiments are required to elucidate the clinical relevance of this study and the possible implications of the extensive use of antimicrobials in oral care.

## Materials and methods

### Strains and culture

The synthetic community was assembled by 14 oral bacterial strains, eight commensal (*Streptococcus sanguinis* LMG14657, *Streptococcus salivarius* TOVE-R, *Streptococcus gordonii* ATCC 49818, *Streptococcus mitis* DSM 12643, *Streptococcus oralis* (clinical isolate), *Actinomyces naeslundii* ATCC 51655, *Actinomyces viscosus* DSM 43327 and *Veillonella parvula* DSM 2007) and six pathobionts (*Porphyromonas gingivalis* ATCC 33277, *Fusobacterium nucleatum* ATCC10953, *Aggregatibacter actinomycetemcomitans* ATCC 43718, *Prevotella intermedia* ATCC 25611, *Streptococcus mutans* ATCC 25175 and *Streptococcus sobrinus* ATCC 33478). The strains were maintained on blood agar No2 (Oxoid, Hampshire,UK) supplemented with hemin (5 mg/mL) (Sigma Aldrich, Belgium), menadione (1 mg/mL) (Sigma Aldrich, Belgium) and 5% sterile horse blood or cultured in liquid medium in Brain Hearth Infusion (BHI) (Roche, Belgium) broth under anaerobic (80% N2, 10% H2, and 10% CO2) conditions.

BHI medium was used for the assembled synthetic community. This medium is enriched with 2.5 g/L Mucin from porcine stomach type III (Sigma, Diegem, Belgium), 1.0 g/L Yeast extract (Oxoid, Hampshire, UK), 0.1 g/L cysteine (Merck—Calbiochem), 2.0 g/L sodium bicarbonate (Sigma Aldrich, Belgium), 0.25% glutamic acid (Merck—Calbiochem), 5.0 mg/L hemin (Sigma Aldrich, Belgium), 1.0 mg/L menadione (Sigma Aldrich, Belgium).

### Minimum inhibitory concentration

The MIC of CHX for each individual strain was evaluated by absorbance. More specifically, 10^7^ cells/mL of each strain was inoculated in serial dilutions of CHX and incubated for 24 h under anaerobic conditions. Following OD_600_ was measured by microtiter plate reader (Tecan Infinite M200 Pro; Tecan UK, Reading, UK).

### Tongue microbiome sampling

The tongue microbiome samples originated from four individuals. The individuals were in good systemic health and had not received antibiotics over the previous 3 months. The donors were asked to refrain from food intake or tongue/tooth brushing for at least 2 h before collection of the tongue biofilm.

Tongue biofilm was sampled by scraping the tongue surface with a disposable tongue scraper (Jordan, Norway) three times from back to front of the tongue dorsum. The scrapped biofilm was suspended in 5 mL of reducing phosphate buffer (8.8 g/L K_2_HPO_4_, 6.8 g/L KH_2_PO_4_ and 1 g/L C_2_H_3_O_2_SNa (Sigma Aldrich, Belgium)) and homogenized by pipetting keeping individual samples separate. One milliliter of each sample was used to inoculate a separate anaerobic penicillin bottle with 15 mL BHI 2 medium. Samples were allowed to separately grow in planktonic conditions for 48 h before used further to grow a biofilm.

### Biofilm model

After the 14 strains had grown individually in BHI broth as described above, the synthetic community was assembled by adding equal concentrations of each strain in BHI 2 medium. Then they were allowed to grow for 48 h under anaerobic (80% N_2_,10% H_2_, and 10% CO_2_) conditions. Subsequently inoculum from the planktonic bioreactor was used to grow biofilms on hydroxyapatite (HA) disks (0.5 inch diameter × 0.04–0.06 inch thick, VWR, Belgium) that were placed in an active attachment biofilm model^42^. Sample of the bioreactor was diluted 1:2 v/v in fresh BHI 2 medium. Two milliliter of the new culture containing the community were added in each well of a 24-well plate and incubated for 24 h at 37 °C in anaerobic conditions and shaking at 170 rpm. Twenty-fours hours-biofilms were used for the following experiments.

The same procedure was followed with the donor samples that had been previously grown in BHI 2 for 48 h.

### Treatment

The biofilms were treated with CHX by placing the HA disks for 5 min in a new 24-well plate with 0.12% CHX (Chlorohexidine digluconate, Sigma Aldrich, Belgium). Non-treated biofilms underwent the same procedure in sterile PBS (Phosphate Buffer Saline, Sigma Aldrich, Belgium). They were subsequently washed twice with PBS and finally placed back in a new 24-well plate with fresh BHI 2 medium. The biofilms were treated every 24 h and samples were collected before and after treatment. Biofilms were disrupted by Trypsin-EDTA 0.05% at 37 °C, 350 rpm for 45 min and then further placed in a sonication bath (37 kHz sonication frequency) for 32 min to disrupt the aggregates.

### Flow cytometry

Samples of the disrupted biofilm were diluted in sterile PBS and stained with the nucleic acid stain SYBR® Green I and Propidium Iodide that stains permeabilized cells^43^. SYBR Green I (10,000× concentrate in DMSO, Invitrogen) was diluted 100 times in 0.22 µm-filtered DMSO (IC Millex, Merck, USA) and Propidium Iodide (20 mM in dimethyl sulfoxide (DMSO), Invitrogen, USA) was diluted 50 times. Samples were stained with 10 µL/mL staining solution. Next, they were incubated in 37 °C for 13 min. All samples were measured with a benchtop Accuri C6+ cytometer (BD Biosciences, Belgium). The stability of the instrument was controlled daily using CS&T RUO beads (BD Biosciences, Belgium) and checking for each measurement the stability of FL1 over time. The blue laser (488 nm)was used for the excitation of the stains. The filters for the (fixed gain) photomultiplier detectors used during the measurements were 533 nm with a bandpass of 30 nm for the green fluorescence (FL-1) and 670 nm longpass filter for the red fluorescence (FL-3). The threshold was set on the 533/30 nm (FL-1) detector at the arbitrary unit of 500. FlTC-A ~ PerCP-A density plots were used to draw gates for intact, damaged and dead cells according to Supplementary Fig. 1.

### DNA extraction and vitality qPCR of the 14-strain biofilms

The disrupted biofilms were treated with PMAxx Dye (Biotium, USA) before further manipulation. DNA extraction was performed by means of bead beating with a PowerLyzer instrument (Qiagen, Venlo, Netherlands) and phenol/chloroform extraction. The surviving bacteria were quantified by vitality qPCR using specific primers^44,45^. The qPCR measurements were performed with a CFX96 Real-Time System (Bio-Rad, Temse, Belgium). In short, Taqman 5′ nuclease assay PCR method was used for detection and quantification of every one of the 14 strains using strain specific primers and probes (Supplementary Table 1). Quantification was based on a plasmid standard curve.

### DNA extraction and amplicon sequencing of multispecies biofilms

DNA from the multi-species biofilms was extracted using the ZymoBIOMICS DNA Microprep Kit (Zymo Research, USA) according to the manufacturer's instructions. 10µl genomic DNA extract was send out to LGC genomics GmbH (Berlin, Germany) for library preparation and sequencing on an Illumina Miseq platform with v3 chemistry with the primers 341F (5′-CCT ACG GGN GGC WGC AG -3′) and 785Rmod (5′-GAC TAC HVG GGT ATC TAA KCC-3′)^46^.

The average number of raw reads per sample was 45712. Read assembly and cleanup was largely derived from the MiSeq SOP described by the Schloss lab. In brief, mothur (v.1.40.3) was used to assemble reads into contigs, perform alignment-based quality filtering (alignment to the mothur-reconstructed SILVA SEED alignment, v. 123), remove chimeras, assign taxonomy using a naïve Bayesian classifier^47^ and SILVA NR v132 and cluster contigs into OTUs at 97% sequence similarity. All sequences that were classified as Eukaryota, Archaea, Chloroplasts and Mitochondria were removed. Also, if sequences could not be classified at all (even at (super)Kingdom level) they were removed. After the above-mentioned filtering, the average reads per sample were 27940. For each OTU representative sequences were picked as the most abundant sequence within that OTU.

### Metabolic activity

Organic acids in the samples were measured with 761 Compact Ion Chromatograph (Metrohm, Switzerland) with a Metrosep Organic acids 250/7.8 column and a Metrosep Organic acids Guard/4.6 guard column. The eluent consists of 1 mM H_2_SO_4_ at a flow rate of 0.8 mL/min. The production of organic acids was calculated as the concentration detected in the liquid that the biofilm was grown normalized for the organic acid concentrations detected in sterile BHI 2 medium.

### Data analysis/statistics

All statistical analysis was performed in R (v3.6.0). The OTU contingency table was imported in R. OTUs with no more than one read in every sample (singletons) were removed^48^. The average number of reads per sample after removing singletons was 27866, while the total number of OTUs was 2350. The graphs representing the 20 most relative abundant genera were generated using the *phyloseq* package^49^ in R (v3.6.0). The taxonomic β-diversity was calculated based on Bray-Curtis dissimilarity index, using the ordinate function of *phyloseq* package, and displayed in a non-metric multidimensional scaling (nMDS) plot.

### Ethics statement

The sampling of human tongue biofilm was approved by the the Medical Ethical Committee of Ghent University with reference number B670201629302. All participants gave their written informed consent prior to their inclusion in the study.

### Reporting summary

Further information on research design is available in the Nature Research Reporting Summary linked to this article.

## Supplementary information

Supplementary Information

Reporting Summary

## Data Availability

Sequences are available on the NCBI Sequence Read Archive (SRA) under accession number PRJNA554992.
