# Supplementary material for: Oral biofilms exposure to chlorhexidine results in altered microbial composition and metabolic profile
Source: NPJ Biofilms Microbiomes. 2020 Mar 20;6:13. doi: 10.1038/s41522-020-0124-3 (PMC7083908; doi:10.1038/s41522-020-0124-3)
Supplement: Supplementary file 1 — Supplementary Information [file 41522_2020_124_MOESM1_ESM.pdf]

Supplementary information

Title:

Oral biofilms exposure to chlorhexidine results in altered microbial composition and metabolic profile

Authors:

Chatzigiannidou Ioanna[1], Teughels Wim[2], Van de Wiele Tom[1], Boon Nico[1]*

Affiliation

[1] Center for Microbial Ecology and Technology, Coupure Links 653, B-9000 Gent

[2] Department of Oral Health Sciences, KU Leuven, Kapucijnenvoer 33, 3000 Leuven, Belgium

*To whom correspondence should be addressed: nico.boon@ugent.be

Supplementary Material and Methods

Supplementary Table 1: A list of the strain specific primers and probes that were used for targeted vitality qPCR

Strain	Target gene		Sequence (5' -> 3')
Prevotella intermedia	16S rRNA	Forward	CGG TCT GTT AAG CGT GTT GTG
		Reverse	CAC CAT GAA TTC CGC ATA CG
		Probe	TGG CGG ACT TGA GTG CAC GC
Porphyromonas gingivalis	16S rRNA	Forward	GCG CTC AAC GTT CAG CC
		Reverse	CAC GAA TTC CGC CTG C
		Probe	CAC TGA ACT CAA GCC CGG CAG TTT CAA
Fusobacterium nucleatum	16S rRNA	Forward	GGA TTT ATT GGG CGT AAA GC
		Reverse	GGC ATT CCT ACA AAT ATC TAC GAA
		Probe	CTC TAC ACT TGT AGT TCC G
Aggregatibacter actinomycetemcomitans	16S rRNA	Forward	GAA CCT TAC CTA CTC TTG ACA TCC GAA
		Reverse	TGC AGC ACC TGT CTC AAA GC
		Probe	AGA ACT CAG AGA TGG GTT TGT GCC TTA GGG
Streptococcus mutans	gftB	Forward	GCC TAC AGC TCA GAG ATG CTA TTC T
		Reverse	GCC ATA CAC CAC TCA TGA ATT GA
		Probe	TGG AAA TGA CGG TCG CCG TTA TGA A
Streptococcus sobrinus	gftT	Forward	TTC AAA GCC AAG ACC AAG CTA GT
		Reverse	CCA GCC TGA GAT TCA GCT TGT
		Probe	CCT GCT CCA GCG ACA AAG GCA GC
Veillonella parvula	16S rRNA	Forward	GAC GAA AGT CTG ACG GAG CA
		Reverse	TGC CAC CTA CGT ATT ACC GC
		Probe	AGC TCT GTT AAT CGG GAC GAA AGG C
Actinomyces viscosus	16S rRNA	Forward	GTG AAG GAG CCA GCT TGC TGG TTC TG
		Reverse	CGG AAC AAA CCT TTC CCA GGC
		Probe	ATG AGT GGC GAA CGG GTG AGT AAC
Actinomyces naeslundii	unknown protein	Forward	TCG AAA CTC AGC AAG TAG CCG
		Reverse	AGA GGA GGG CCA CAA AAG AAA
		Probe	GGG TAC TCT AGT CCA AAC TGG CGG ATA GCG
Streptococcus oralis	gftR	Forward	ACC AGC AGA TAC GAA AGA AGC AT
		Reverse	AGG TTC GGG CAA GCG ATC TTT CT
		Probe	AAG GCT GCT GTT GCT GAA GAA GT
Streptococcus sanguinis	gftP	Forward	CAA AAT TGT TGC AAA TCC AAA GG
		Reverse	GCT ATC GCT CCC TGT CTT TGA
		Probe	AAA GAA AGA TCG CTT GCC AGA ACC GG
Streptococcus salivarius	dextranese	Forward	AAC GTT GAC CTT ACG CTA GC
		Reverse	ACC GTA ACG TGG GAA AAC TG
		Probe	GTA GCG TCA GAG TGG TTG AC
Streptococcus mitis	16S rRNA	Forward	GGC TCG TAG TCT GGA GAT GG
		Reverse	TAG GTC GTC GTC CCA AGG AA
		Probe	CGA AGA GCA CCA ATA GCA CCT CCC
Streptococcus gordonii	gftG	Forward	CGG ATG ATG CTA ATC AAG TGA CC
		Reverse	GTT AGC TGT TGG ATT GGT TGC C
		Probe	AGA ACA GTC CGC TGT TCA GAG CAA

Supplementary Table 2: A list of Minimum Inhibitory Concentration (MIC) of chlorhexidine for each individual strain that was used in the 14 strain biofilm consortium.

Strain	MIC (µg/mL)
Prevotella intermedia	1.95
Porphyromonas gingivalis	1.95
Fusobacterium nucleatum	1.95
Aggregatibacter actinomycetemcomitans	1.95
Streptococcus mutans	3.9
Streptococcus sobrinus	1.95
Veillonella parvula	3.9
Actinomyces viscosus	1.95
Actinomyces naeslundii	1.95
Streptococcus oralis	7.8
Streptococcus sanguinis	3.9
Streptococcus salivarius	1.95
Streptococcus mitis	1.95
Streptococcus gordonii	7.8

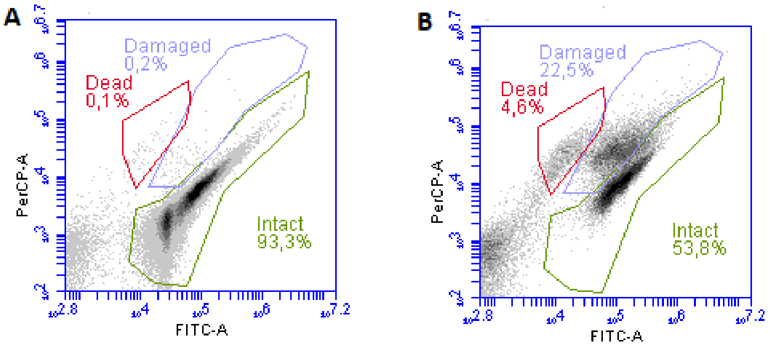

Supplementary Figure 1: Gating Strategy for Flow cytometry. The samples were stained with SYBR Green and Propidium Iodide and the gating was on density plots on FITC-A (green) ~ PerCP-A (red) channel according to the figure – two different samples are exhibited: A. non-treated sample with highest intact cell concentration and B. sample after CHX treatment
